# Fluorescent Probe Combined with Photoelectric Analysis Technology for Detection of *Escherichia coli*

**DOI:** 10.3390/bios13020150

**Published:** 2023-01-18

**Authors:** Qian Cui, Yongjie Zhong, Wenkai Shang, Fuming Deng, Buhua Wang, Jiajia Wu, Peng Wang, Liudang Wan, Keling Wang, Lingchen Fang, Rui Dai, Han Zhang, Rodrigo Ledesma-Amaro, Yunuo Zhang, Jiaomei Huang

**Affiliations:** 1 State Key Laboratory of Marine Resource Utilization in South China Sea, Hainan University, Haikou 570228, China; 2 Coconut Research Center, Coconut Research Institute, Chinese Academy of Tropical Agricultural Science (CATAS), Haikou 570228, China; 3 CAS Key Laboratory of Marine Environmental Corrosion and Bio-Fouling Institute of Oceanology, Chinese Academy of Sciences, Qingdao 266071, China; 4 Insititue of Biotechnology, ViewKr, Haikou 570228, China; 5 Department of Bioengineering and Imperial College Centre for Synthetic Biology, Imperial College London, London SW7 2AZ, UK

**Keywords:** *Escherichia coli*, fluorescent probe, photoelectric analysis, microbial photoelectric detection system, Hainan coconut water

## Abstract

Food safety is facing great challenges in preventing foodborne diseases caused by pathogenic pollution, especially in resource-limited areas. The rapid detection technique of microorganisms, such as immunological methods and molecular biological methods, plays a crucial key in timely bioanalysis and disease treatment strategies. However, it is difficult for these methods to simultaneously meet the criteria of simple operation, high specificity, and sensitivity, as well as low cost. Coconut water is known as the “water of life” in Hainan. It is a refreshing and nutritious beverage which is widely consumed due to its beneficial properties to health. Coconut water processing is an important pillar industry in Hainan. The detection of pathogenic microorganisms, such as *Escherichia coli*, in coconut water has become an important factor which has restricted the upgrading and development of this industry. Based on the needs of industrial development, we developed a microbial photoelectric detection system which was composed of a fluorescent probe detection reagent and a photoelectric sensor detection device. This system combined microbial enzyme targets, selective fluorescent substrate metabolism characteristics, and a photoelectric sensor signal transduction mechanism, which produce a strong signal with a high signal-to-noise ratio. The microbial detection system developed here has a simple structure, simple and convenient operation, short detecting time (≥2 h), and high sensitivity (1 CFU/mL). This system may also enable early warning and monitoring programs for other pathogenic microorganisms in order to promote the overall competitiveness of the Hainan coconut water industry.

## 1. Introduction

Pathogenic microorganisms, such as parasites, bacteria, or viruses, pose a significant threat by transmission to the public through contaminated meat, vegetables, or beverages [1]. Common foodborne pathogens, including Escherichia coli, Staphylococcus aureus, Bacillus, Listeria monocytogenes, Salmonella, Yersinia pseudotuberculosis, Campylobacter, and so on [2,3], cause huge economic losses. For instance, it has been estimated that there are 700,000 diseases in the Netherlands each year, of which the burden of foodborne diseases kills at least 3800 disability-adjusted lives and leads to EUR 65 million in costs per year [4]. In addition, there are an estimated 1,500,000 foodborne diseases caused by known/unknown pathogens in the UK each year according to the statistics [5,6]. Therefore, it is urgent to develop a sensitive signal reporter with a one-step procedure that can be employed in pathogen diagnosis [7].

*Escherichia coli* is one of the most well-adapted and pathogenically versatile bacterial organisms. It causes a variety of human infections, including gastrointestinal illnesses and extraintestinal infections. It is also part of the intestinal commensal flora of humans and other mammals. The presence of *E. coli* not only affects the quality and taste of coconut water but may also become a threat to human health [8,9,10].

At present, methods for the detection of pathogens mainly include microbial culture [11,12], immunological detection [13,14,15], molecular biological-based techniques [15,16,17], metabolomics technology [15,18,19], and the use of biosensors [15,20]. Among them, the methods based on microbial cultivation rely on the physical and chemical characteristics of each bacterium. This method displays a low number of false positives, and it is simple and easy to observe, but it is time-consuming and has low efficiency [12]. Immunology methods are based on a specific recognition between antigen and antibody, and they are the most widely used methods in food pathogen detection. This method is economical and practical, but it has a high degree of cross-reaction [13]. Molecular biological methods include nucleic acid hybridization, gene recombination, PCR technology, etc., which are often suitable for the detection of uncommon or new pathogenic microorganisms. These methods are rapid, sensitive, and specific, but they require a high level of experimental design or complicated operational procedures [16,21,22,23,24]. Metabolomics is a high-resolution analytical method that obtains detailed information on various metabolites produced in biological systems. However, significant consideration is needed for the selectivity and sensitivity of this approach, and aspects such as metabolite recognition and annotation often remain a challenge [18,25,26].

Biosensor methods based on small-molecule fluorescent probes are of interest because of their high sensitivity and selectivity, as well as their potential for automated detection. Fluorescent probes are useful in targeting particular enzymes of interest, such as proteases and caspases [27,28]. Fluorescent probes targeting enzymes are small molecular compounds that can react with specific enzymes and emit fluorescence. These compounds can accurately and rapidly bind or react with specific regions of the targeted enzymes [29,30,31,32].

In addition, photoelectric biosensors—devices that can sense and convert various measured signals—have been developed and improved for over a decade [33]. Due to their small size, high sensitivity, and high precision, nanosensors have attracted extensive attention in recent years [34,35], and they are used in numerous fields, especially for the detection of pathogenic microorganisms [1,36,37,38,39].

In this study, we created a microbial photoelectric detection system that combines a fluorescent probe with a photon-sensing technology to monitor *Escherichia coli* via fluorescence and color changes caused by microbial metabolism using a fluorescence photoelectric detection system. The instrument is suitable for the detection of *E. coli*, Salmonella, and other pathogenic microorganisms. The system was developed to analyze data in real time and provide an early warning for the control of pathogenic microorganisms.

## 2. Materials and Methods

### 2.1. Materials and Chemicals

The strains used in the experiment were purchased from the National Culture Preservation Center: *Escherichia coli* (CICC 10389), *Staphylococcus aureus* (CICC 10384), *Salmonella typhi* (CICC 21484), and *Pseudomonas aeruginosa* (CICC 21625). The coconut water samples were obtained from Coconut Research Institute, Chinese Academy of Tropical Agricultural Sciences.

### 2.2. Microbial Photoelectric Detection System

**Figure 1:**
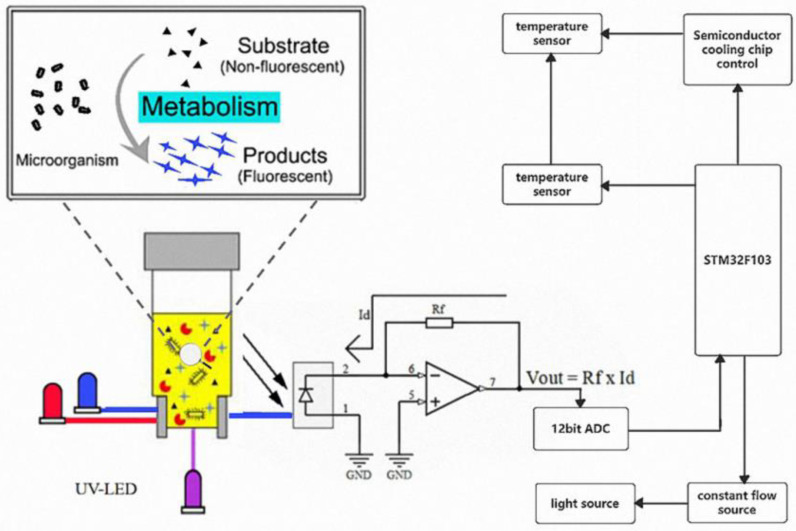
Overall design block diagram of microbial photoelectric detection system.

The microbial photoelectric detection system is an analytical instrument that integrates microbial culture medium and detection system. The microbial photoelectric detection system consists of four modules based on different hardware functions (Figure 1), including a main control module, light source module, temperature control module, and detection module. The temperature control module provides the most suitable incubation temperature for the microbial tube. Fluorescence or colorimetric detection is then performed based on the metabolic characteristics of microorganisms during their growth and the corresponding signaling molecules. After the microbial photoelectric detection system is assembled, accuracy verification is performed between different channels of the instrument and between different instruments to ensure the accuracy of the instrument.

### 2.3. Optimization Method

A series of experiments were used to optimize the type and concentration of inhibitors in the medium formulation to improve the specificity of the method. Firstly, by comparing the inhibitory ability of multiple inhibitors on different strains, suitable inhibitors were screened. The concentration of each component in the medium was then determined using orthogonal experiments. At the same time, the plate method was used to confirm the culture concentration in each experiment to ensure the suitability of the medium formulation.

### 2.4. Bacterial Detection with Microbial Photoelectric Detection System

**Figure 2:**
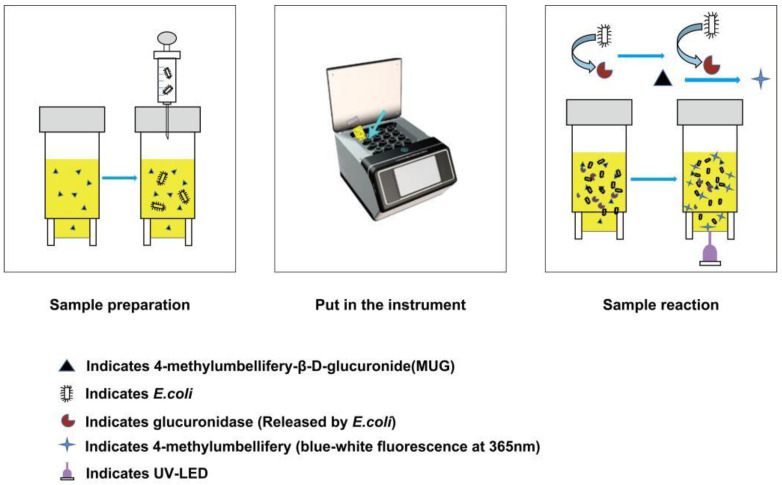
Detection process and principle schematic diagram of the microbial photoelectric detection system.

Different coconut water samples containing 0–10^6^ CFU/mL of bacteria were prepared. The coconut water samples were obtained from Coconut Research Institute, Chinese Academy of Tropical Agricultural Sciences. The bacterial detection method was as follows: First, the initial bacterial solution was prepared, and then the bacterial solution was gradient diluted with sterilized coconut water. Then, 1 mL of sample was injected into the tube with a sterile syringe, shaken well, placed in a microbial photoelectric detection system, and programmed to 37 °C for 16 h (Figure 2). At the same time, 1 mL of samples of each concentration was taken and plate-coated. Each test was performed in parallel and was repeated twice.

## 3. Results and Discussion

### 3.1. Verification of the Microbial Photoelectric Detection System

The microbial photoelectric detection system quantifies microorganisms by detecting the light signal generated by the measured sample during growth. In this study, we aimed to develop a multichannel biosensor for the simple, rapid, and highly sensitive detection of *Escherichia coli*.

The working principle of the microbial photoelectric detection system can be summarized as follows: After culturing for a period of time in the microbial photoelectric detection system, the glucuronidase produced by *Escherichia coli* will hydrolyze the β-uronic acid bond of 4-methylumbellifery-β-D-glucuronide (MUG), which releases 4-methylumbelliferone to produce blue-white fluorescence under a 365 nm ultraviolet lamp (Figure 2) [40,41]. Theoretically, the higher the concentration of the pathogen of interest in the sample, the shorter the time to detect the fluorescence. Finally, the inflection point time of detected fluorescence and the corresponding amount of bacteria are measured to quantitatively detect *Escherichia coli*.

**Figure 3:**
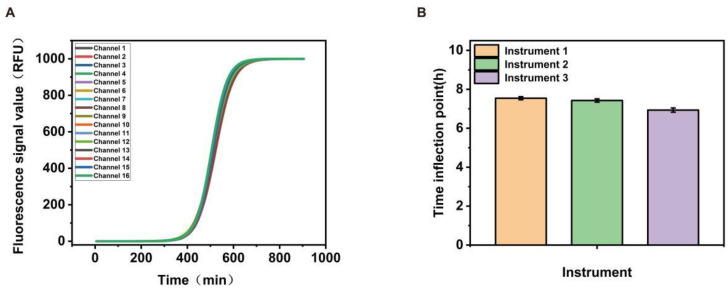
(**A**) shows the consistency among the various channels of the instrument; (**B**) shows the differences with different instruments.

As the microbial photoelectric detection system consists of 16 channels, we tested and compared the light source consistency of each channel to ensure the accuracy and repeatability of the test results. As shown in Figure 3A, we tested different channels of the instrument using the same concentration of *E. coli* solution of 10^3^ CFU/mL, and a good consistency was obtained between the different channels. As there was more than one microbial photoelectric detection system used in the experiment, we selected three of them for comparison, as shown in Figure 3B, and although there were slightly different outputs, all were within acceptable limits.

### 3.2. Optimization Results

**Figure 4:**
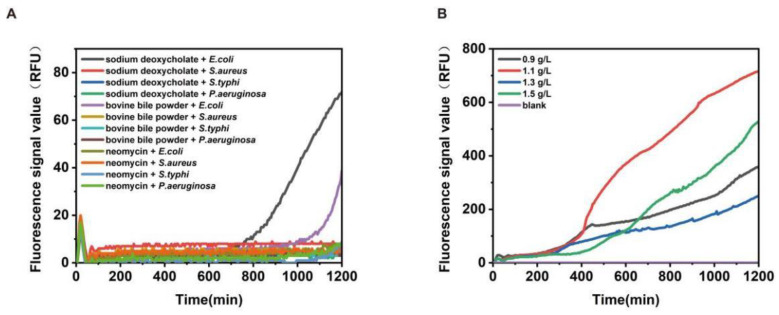
Optimization of media formulations. (**A**) shows that sodium deoxycholate works best. (**B**) shows that sodium deoxycholate had the lowest inhibitory effect on *E. coli* at 1.1 g/L.

In this study, we optimized components in the medium to ensure the specificity of the method. First, we searched for selected inhibitors, including sodium deoxycholate, bovine bile powder, and neomycin [42,43,44]. It was found that sodium deoxycholate could inhibit miscellaneous bacteria well and had the weakest inhibitory effect on the target strain (Figure 4A). In the next step, we optimized certain experimental conditions, including the concentration of sodium deoxycholate. In this regard, different concentrations of sodium deoxycholate were used in the media formulations, and the experimental results showed that there was no curve growth in the blank test without *E. coli*, and the shape of the growth curve of *E. coli* was the best when the concentration of sodium deoxycholate was 1.1 g/L (Figure 4B). The best combination in these tests was determined to be the final formulation of *Escherichia coli*.

**Figure 5:**
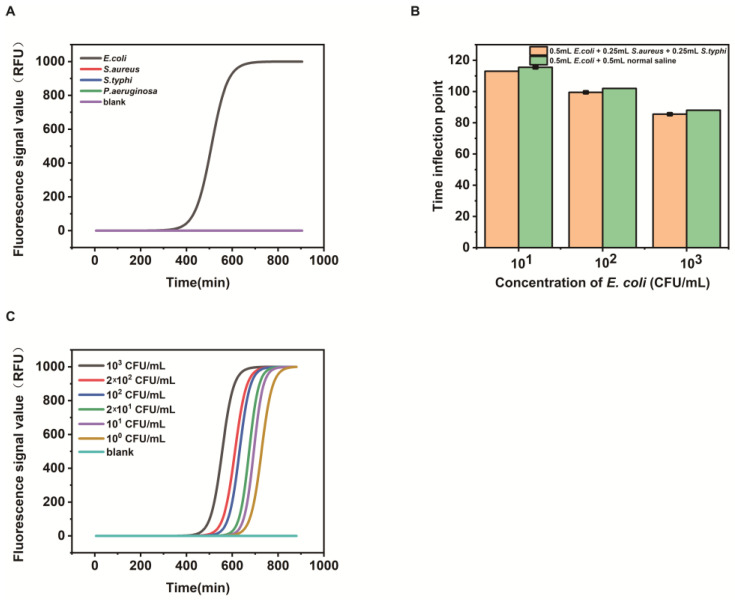
Performance validation of media formulations. (**A**) shows good specificity of the formulation; (**B**) indicates that the medium is capable of detecting *E. coli* without interference from other bacteria, even in the presence of bacteria; (**C**) shows that this medium is capable of detecting 1 CFU/mL of *E. coli*.

We tested the performance of this formulation using the microbial photoelectric detection system. As shown in Figure 5A, *E. coli* showed a strong fluorescence signal compared to *S. aureus*, *S. typhi*, and *P. aeruginosa*, while the other three bacteria had little to no observable fluorescence signal, indicating good specificity of the assay. To further verify the anti-interference properties of the modified formulation, *E. coli* was mixed with *S. aureus* and *S. typhi*. As shown in Figure 5B, the other bacteria did not interfere, which again indicates that the assay has good specificity. As shown in Figure 5C, the minimum detection limit of *E. coli* reached 1 CFU/mL, indicating the high sensitivity of this detection method.

### 3.3. Evaluation of the Microbial Photoelectric Detection System Technology

**Figure 6:**
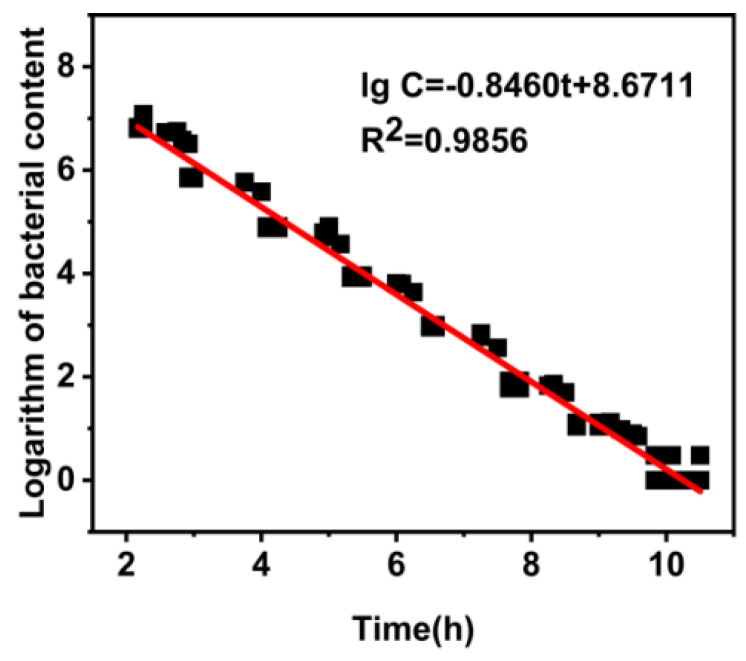
Standard curve equation for detection of *E. coli* in coconut water using the microbial photoelectric detection system.

In order to obtain the standard curve equation for the detection of *E. coli* in coconut water, we selected a bacterial solution with an *E. coli* concentration of 10^6^–10^−1^ CFU/mL after dilution to conduct the experiment. The data obtained from the above tests were plotted with the time (h) as the horizon coordinate and the logarithm of bacterial count (CFU/mL) −lg CFU/mL as the vertical coordinate, and the generated standard curve equation was lg C = −0.8460 t + 8.6711 (lg C is lg CFU/mL). The R^2^ of the standard curve equation is 0.9856 (Figure 6), which is close to 1, indicating that the linear fitting of the equation is good, and it can be used as the standard equation.

Under optimized conditions, the ultra-sensitive and quantitative detection capabilities were verified. Two sets of *E. coli* solutions of different concentrations were prepared using the gradient dilution method, and for comparison, two sets of experiments were evaluated according to the national standards [45,46]. In addition, samples in both groups used two instruments to evaluate the consistency and reproducibility of the method. The results obtained using the national standard method for gradient dilution and our microbial photoelectric detection system, shown in Table 1 (the acceptance criteria are referring to GB/T 6379, ISO16140, and AOAC), demonstrated that both methods meet the acceptance standard.

**Figure 7:**
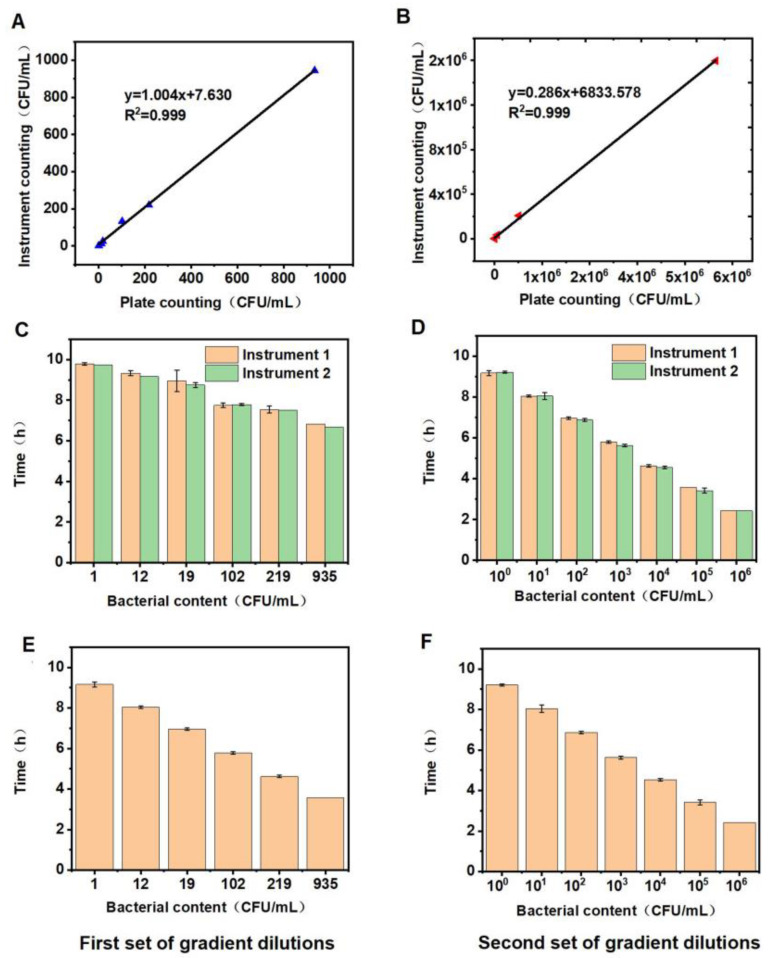
Detection results of two groups using microbial photoelectric detection system and national standard method. (**A**,**B**) are correlation analysis comparisons of the test results between the two groups using national standard method and microbial photoelectric detection system, respectively. (**C**,**D**) are the consistency analyses of different microbial photoelectric detection system instruments using group 1 and group 2 diluted solutions, respectively. (**E**,**F**) are reproducibility analyses of the results of the two groups ((**E**) is for group 1, and (**F**) is for group 2) using microbial photoelectric detection system.

In addition, we conducted a linear comparison on the bacterial counts measured by the two methods. As addressed in Figure 7A,B, the specific linear relationship showed that both of the correlation coefficients were 0.999. This results, once again, validated that the microbial photoelectric detection system measurements meet the national standard. The instrument consistency measurement with different microbial photoelectric detection systems was also assessed (Figure 7C,D), and a desired result was achieved. We also observed a good reproducibility result, as shown in Figure 7E,F. Thus, we can conclude that the microbial photoelectric detection systems show good consistency and reproducibility at low and high concentrations, which make them suitable for the detection of *E. coli* in coconut water.

At the same time, we compared the microbial photoelectric detection system with the standard plate counting method in aspects including the detection time, assay procedure, flux, and cost (Table 2). A comprehensive analysis shows that the microbial photoelectric detection system has obvious advantages in all aspects of performance.

### 3.4. Method Assessment in Real Sample

**Figure 8:**
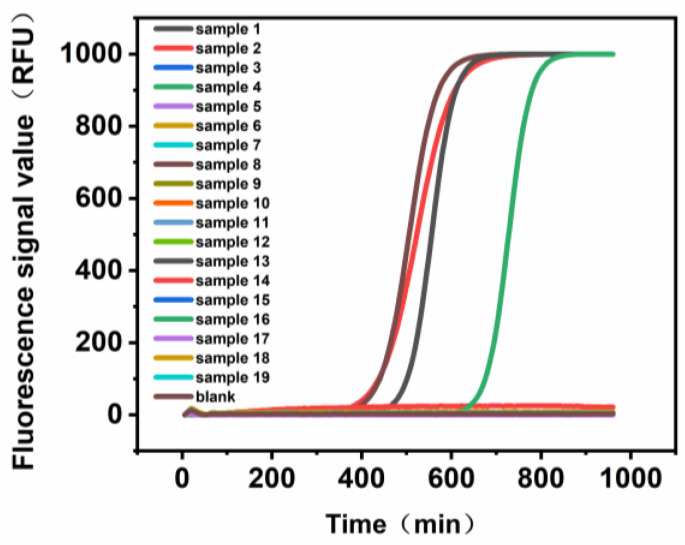
Test results of the actual sample.

We validated the ability of the microbial photoelectric detection system to detect *E. coli* in coconut water using 19 real samples. As shown in Figure 8, samples 1, 2, 8, and 16 are positive, while the rest of the samples are negative. At the same time, the number of *E. coli* colonies obtained using plate count when diluting the actual sample (Table S1 in the Supplementary Material) was 10^6^ CFU/mL, which is in the same order of magnitude as from the microbial photoelectric detection system method. Therefore, it can be concluded that the microbial photoelectric detection system can be used to, in fact, detect *E. coli* in coconut water.

## 4. Conclusions

In this study, we developed a microbial photoelectric detection system to detect Escherichia coli in coconut water. The standard curve was established according to the plate counting method, which was performed in accordance with the national standard.

The standard curve lgC = −0.8460 t + 8.6711 was established and verified through a series of experiments. The results showed that the national standard method for coating plate detection and the microbial photoelectric detection system were consistent, suggesting a high accuracy rate of this method. In addition, when different instruments were used for testing, good consistency and reproducibility were achieved. The results of specificity testing showed that *Escherichia coli* could be detected in coconut water without interference when it was mixed with other bacteria (*Staphylococcus aureus*, *Salmonella typhi*, and *Pseudomonas aeruginosa*). Moreover, the instrument was highly sensitive, with a LOD of 1 CFU/mL achieved. When a real coconut water sample was used, the detection capacity of the microbial photoelectric detection system was in the same order of magnitude as the result calculated from the MUG plate count, indicating that the microbial photoelectric detection system can precisely detect *Escherichia coli* in coconut water with high reference significance.

Comparing four commonly used microbial detection technologies, Multiskan™ FC, QPCR, ATP Portable Fluorescent Biochemical Rapid Analyzer, and Easicult Combi, the microbial photoelectric detection system achieved higher sensitivity than the ATP Portable Fluorescent Biochemical Rapid Analyzer (100 CFU/mL) and Easicult Combi (1000 CFU/mL). Despite the current detection throughput being less than that of QPCR (96 samples) and Multiskan™ FC (96 samples),we will continue to optimize according to market demand. It is worth mentioning that this system has a significant advantage in terms of detection cost.

Therefore, we can conclude that the microbial photoelectric detection system can be applied to detect the presence and content of *Escherichia coli* in coconut water with a simpler and faster procedure than the traditional detection methods; it also showed excellent performance in accuracy, repeatability, specificity, and sensitivity. This method can be tuned to enable early warning and monitoring programs for other pathogenic microorganisms in order to promote the overall competitiveness of the Hainan coconut water industry. In the future, we will develop detection systems for *Pseudomonas aeruginosa, Salmonella,* and *Clostridium perfringens* to expand the detection range of pathogenic bacteria.

In the future, we will continue to improve and optimize the microbial photoelectric detection system according to market demand, so that it can play a valuable role in microbial detection, including food safety, human public health, nutrition, and health and disease prevention.

## Figures and Tables

**Table 1:** Acceptance results of two groups obtained using national standard method and microbial photoelectric detection system.

Parameters	Acceptance Standard	Group 1 Acceptance Results	Group 1 Conclusions	Group 2 Acceptance Results	Group 2 Conclusions
accuracy	≥95%	100%	qualified	100%	qualified
sensitivity	≥95%	100%	qualified	100%	qualified
specificity	≥98%	100%	qualified	100%	qualified
false negative	<5%	0%	qualified	0%	qualified
false positive	<0%	0%	qualified	0%	qualified

**Table 2:** Performance comparison between microbial photoelectric detection system and standard plate count method.

Methods	Detection Time	Assay Procedure	Assay Flux	Test Cost (RMB)
microbial photoelectric detection system	≥2 h	one-step	16 samples	6
standard plate count method	≥24 h	multi-step	1 sample	2–3

## Data Availability

The data sets generated during and/or analyzed during the current study are available from the corresponding author on reasonable request.

## Supplementary Materials

The following supporting information can be downloaded at: https://www.mdpi.com/article/10.3390/bios13020150/s1, Table S1: Method Assessment in Real Sample between Instrument detection and Flat panel detection.
