# Supplementary material for: Fluorescent Probe Combined with Photoelectric Analysis Technology for Detection of Escherichia coli
Source: Biosensors (Basel). 2023 Jan 18;13(2):150. doi: 10.3390/bios13020150 (PMC9953772; doi:10.3390/bios13020150)
Supplement: Supplementary file 1 [file biosensors-13-00150-s001.zip › biosensors-2066575-supplementary.pdf]

Table S1 Method Assessment in Real Sample between Instrument detection and Flat panel detection

Actual sample name	Instrument detection value （CFU/mL）	Flat panel detection value (CFU/mL)
Sample 1	$(0.523\pm0.016)\times10^{2}$	$(0.678\pm0.023)\times10^{2}$
Sample 2	$(0.870\pm0.024)\times10^{3}$	$(0.798\pm0.047)\times10^{3}$
Sample 3	Neg.	Neg.
Sample 4	Neg.	Neg.
Sample 5	Neg.	Neg.
Sample 6	Neg.	Neg.
Sample 7	Neg.	Neg.
Sample 8	$(0.117\pm0.035)\times10^{4}$	$(0.238\pm0.024)\times10^{4}$
Sample 9	Neg.	Neg.
Sample 10	Neg.	Neg.
Sample 11	Neg.	Neg.
Sample 12	Neg.	Neg.
Sample 13	Neg.	Neg.
Sample 14	Neg.	Neg.
Sample 15	Neg.	Neg.
Sample 16	$(0.231\pm0.067)\times10^{0}$	$(0.101\pm0.053)\times10^{0}$
Sample 17	Neg.	Neg.
Sample 18	Neg.	Neg.
Sample 19	Neg.	Neg.
Blank	Neg.	Neg.
